# Supplementary material for: Site‐specific noninvasive delivery of retrograde viral vectors to the brain
Source: Bioeng Transl Med. 2025 Aug 21;11(1):e70062. doi: 10.1002/btm2.70062 (PMC12821222; doi:10.1002/btm2.70062)
Supplement: Supplementary file 1 — FIGURE S1: Ultrasound pressure provides safe and effective FUS‐BBBO. Through intravenous injection of Evans blue dye (EBD), we tested peak pressures on our FUS system at 1.5 MHz frequency, with 1 Hz pulse repetition frequency for 120 pulses. We observed increased BBB opening with the increase in peak pressure without the presence of hemorrhage and tissue damage, with FUS‐BBBO at 1.2 MPa showing both safe and effective extravasation of EBD into the targeted site in 3 out of 4 mice. We performed hematoxylin staining on these sections and found microhemorrhages in 1 out of n = 4 mice, appearing as petechiae within a 600 μm by 1100 μm bounded box. We used relatively few animals for this optimization due to the large amount of prior data in our and other groups on the FUS‐BBBO. The pressures reported follow the instrument manufacturer's calibration. Scale bars are 500 μm. FIGURE S2: Transduction of different cell types in the brain by AAV8 and AVV9.retro. Since we used a neuron‐specific human synapsin promoter to drive expression of mCherry and GFP, and observed no substantial transduction of (a) astrocytes, (b) oligodendrocytes and (c) microglia/macrophages in the brain by either AAV9.retro or AAV8. (d) Neuronal transduction was observed Representative images were obtained from mice (n = 6) co‐injected intravenously with AAV8 (red, mCherry) and AAV9.retro (green, EGFP) at 1010 viral particles per gram of body weight. Sections were imaged on a confocal microscope with a 20× objective counterstained for glial cells (GFAP, violet), oligodendrocytes (Olig2, violet), microglia/macrophages (Iba1, violet) and neurons (NeuN, violet). Scale bars are 100 μm. FIGURE S3: Expression of viral vectors in peripheral tissues. No detectable expression was observed in the (a) kidneys, (b) liver and (c) spleen. Representative images were obtained from mice (n = 8) co‐injected intravenously with AAV8 (red, mCherry) and AAV9.retro (green, EGFP) at 1010 viral particles per gram of body weight [file BTM2-11-e70062-s001.docx]

**Site-Specific Noninvasive Delivery of Retrograde Viral Vectors to the Brain**

**Manwal Harb^1,2^**, **Shirin Nouraein^2,3^, Jerzy O. Szablowski^1,2,3,4,*^**

^1^Department of Bioengineering, Rice University, Houston, TX 77030, USA

^2^Rice Neuroengineering Initiative, Rice University, Houston, TX 77030, USA

^3^Synthetic, Systems, and Physical Biology Program, Rice University, Houston, TX 77005, USA

^4^ Applied Physics Program, Rice University, Houston, TX 77005, USA

**Supplementary Figures**


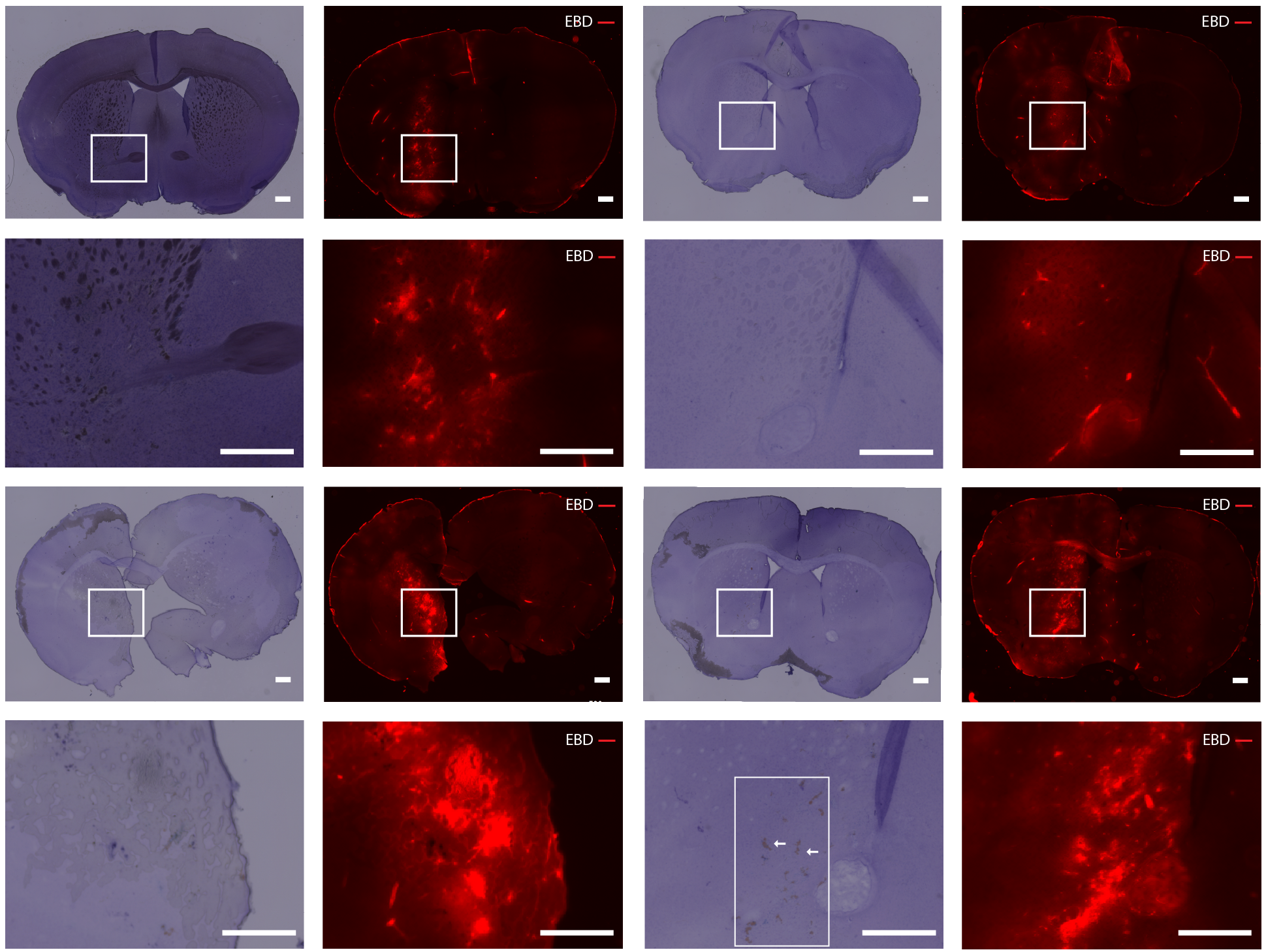


**Supplementary Figure S1. Ultrasound Pressure provides Safe and Effective FUS-BBBO**

Through intravenous injection of Evans Blue Dye (EBD), we tested peak pressures on our FUS system at 1.5 MHz frequency, with 1 Hz pulse repetition frequency for 120 pulses. We observed increased BBB opening with the increase in peak pressure without the presence of hemorrhage and tissue damage, with FUS-BBBO at 1.2 MPa showing both safe and effective extravasation of EBD into the targeted site in 3 out of 4 mice. We performed hematoxylin staining on these sections and found microhemorrhages in 1 out of n=4 mice, appearing as petechiae within a 600-micron by 1100-micron bounded box. We used relatively few animals for this optimization due to the large amount of prior data in our and other groups on the FUS-BBBO. The pressures reported follow the instrument manufacturer’s calibration. Scale bars are 500 μm.


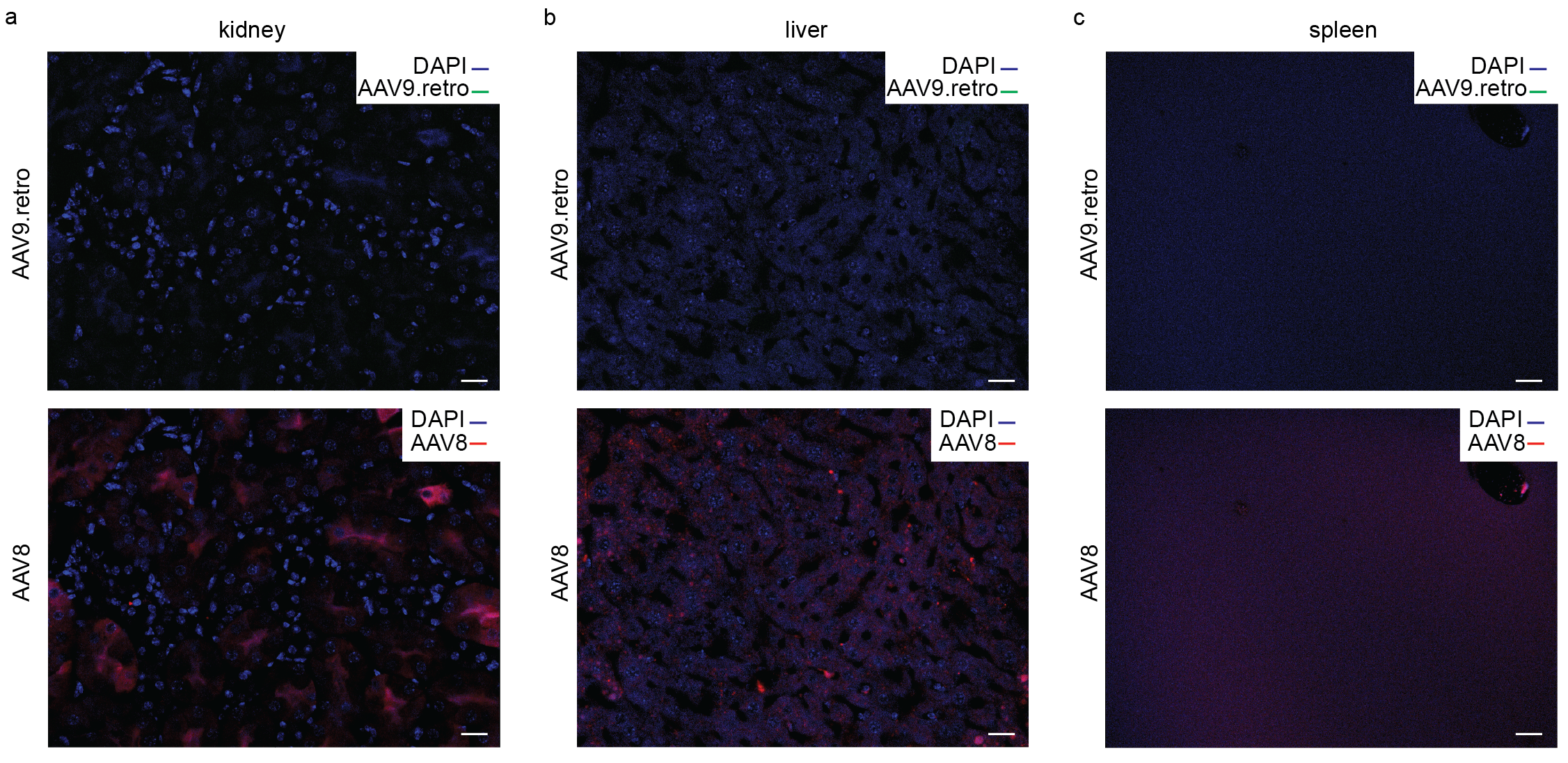


**Supplementary Figure S3. Expression of viral vectors in peripheral tissues.**

No detectable expression was observed in the **a)** kidneys, **b)** liver and **c)** spleen. Representative images were obtained from mice (n=8) co-injected intravenously with AAV8 (red, mCherry) and AAV9.retro (green, EGFP) at 10^10^ viral particles per gram of body weight. Sections were imaged on a confocal microscope with a 20x objective counterstained with a nuclear stain (DAPI, blue). Scale bars are 100 μm.


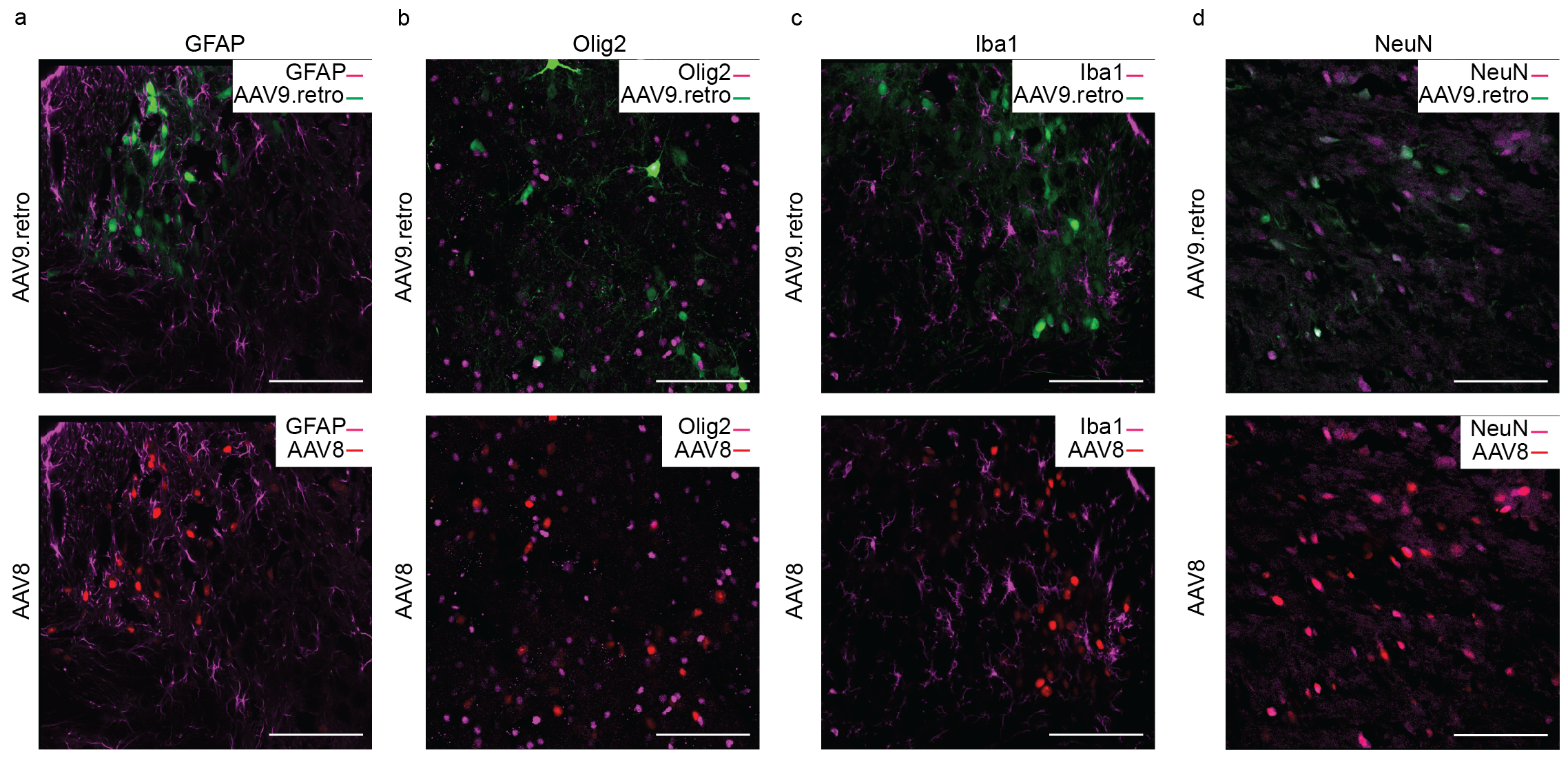


**Supplementary Figure S2. Transduction of different cell types in the brain by AAV8 and AVV9.retro.**

Since we used a neuron-specific human synapsin promoter to drive expression of mCherry and GFP, and observed no substantial transduction of **a)** astrocytes, **b)** oligodendrocytes and **c)** microglia/macrophages in the brain by either AAV9.retro or AAV8. **d)** Neuronal transduction was observed Representative images were obtained from mice (n=6) co-injected intravenously with AAV8 (red, mCherry) and AAV9.retro (green, EGFP) at 10^10^ viral particles per gram of body weight. Sections were imaged on a confocal microscope with a 20x objective counterstained for glial cells (GFAP, violet), oligodendrocytes (Olig2, violet), microglia/macrophages (Iba1, violet) and neurons (NeuN, violet). Scale bars are 100 μm.
